# Treatment Modalities of Pediculosis Capitis: A Narrative Review

**DOI:** 10.7759/cureus.45028

**Published:** 2023-09-11

**Authors:** Rahul Apet, Lekhavardhini Prakash, Kritika H Shewale, Sugat Jawade, Rishi Dhamecha

**Affiliations:** 1 Medicine, Jawaharlal Nehru Medical College, Datta Meghe Institute Of Higher Education and Research, Wardha, IND; 2 Medicine, Jawaharlal Nehru Medical College, Datta Meghe Institute of Higher Education and Research, Wardha, IND; 3 Dermatology, Jawaharlal Nehru Medical College, Datta Meghe Institute of Higher Education and Research, Wardha, IND; 4 Medicine and Surgery, Jawaharlal Nehru Medical College, Datta Meghe Institute of Higher Education and Research, Wardha, IND

**Keywords:** dimethicone, wet comb, head lice, pediculosis capitis, spinosad, permethrin, pediculosis

## Abstract

The review has been done to find out the best-suited treatment modality for pediculosis capitis. Pediatric patients frequently experience pediculosis capitis, a head louse infestation brought on by the Pediculus humanus var. capitis. The primary sign of head lice infestation is a scratchy scalp, and the presence of living nits confirms this diagnosis. When a doctor diagnoses pediculosis as a primary bacterial infection, a bacterial impetignization and secondary infection, and cervical and occipital lymphadenopathy might make the clinical diagnosis more difficult. A proper therapy of pediculosis requires screening and treatment of all close contacts. The careful use of topical pediculicidal treatments, especially permethrin lotion and wet combing with a fine tooth comb, is required for the medical treatment of a head louse infestation.

We’ve tried to outline the key points of treating head lice infestations. There are several treatment alternatives suggested, including over-the-counter permethrin and pyrethrin as well as prescription medications including malathion, lindane, benzyl alcohol, and spinosad.

## Introduction and background

Ectoparasitic infection of the hair and scalp known as pediculosis capitis is a global public health problem, especially in children. The human head louse, or Pediculus humanus var. capitis, which has just one human host, is the cause of the condition [1]. The disease-carrying lice, Pediculus humanus var. capitis De Geer (Anoplura: Pediculidae), feed mostly on blood. Children between the ages of six and twelve are frequently affected by an infestation. In rural regions, the prevalence percentage for children between the ages of three and thirteen ranges from 13.3% to 49%. Especially in rural and poor areas, girls are two to four times more likely to be infected than males because of the length of their hair [2]. Aside from the youngster feeling embarrassed, pediculosis capitis can cause significant social misery, discomfort, parental concern, and unneeded absences from school and employment. The incidence of pediculosis has increased considerably during the past three decades. Additionally, throughout the years, cases of treatment failures brought on by lice that were immune to certain medications have surfaced [3]. In contrast to body lice, which more frequently afflict homeless and displaced people and should be suspected in these populations when there are symptoms of scratching and poor hygiene, and during the colder months of the year, head lice are the most prevalent lice and do not respect social boundaries [4]. The louse is primarily seen living in close proximity to human scalps and is believed to consume blood meals six times each day. Each time a blood meal occurs, a small amount of louse saliva is injected into the scalp skin. Due to the host becoming sensitized to the louse antigen and feces as a result, there is an inflammatory response that results in scratching and eventual impetignization [2]. Clinical characteristics are used to make the diagnosis, and topical 1% permethrin and oral ivermectin are used to treat pediculosis. Wet combing, 0.5% malathion, and more recent medications like spinosad are some more therapeutic options. Once the pediculosis is treated, the pediculid and eczema will ultimately go away. Certain antibiotics may affect or eliminate these bacteria from the louse's gut, body lice that have fed upon the antibiotic-laden blood of people may be burdened and die. The general signs and symptoms of lice in which steroids are given include intense pruritis, general restlessness, and red bumps. On occasion, a brief course of a combination of topical antibiotic and steroid medication may be necessary [5]. The typical criteria for diagnosing head lice infestations include the presence of lice, the presence of eggs attached to hair shafts, scratching, and swelling of the scalp and neck. The "wet combing" method using a nit detection comb is therefore the best way to identify an active infection. Crushing lice after hand or comb removal as well as chemical solutions are some of the various methods used to treat head lice. Lice have been combated with a variety of pediculicides. Organochlorines [lindane, dichlorodiphenyltrichloroethane (DDT)], organophosphates (malathion), carbamates (carbaryl), pyrethrins (pyrethrum), and pyrethroids (permethrin, phenothrin, and bioallethrin) are the most widely used [6]. Changes in pediculicide formulations and incorrect application are additional factors that contribute to resistance. Due to necessary safety modifications in formulations and unforeseen effects of new packaging on chemical components of these goods, the efficacy of pyrethrins has reduced during the past 20 years. Pediculicides made to be applied to moist hair can be too diluted to work as intended. As a cost-saving measure, patients may potentially use too little product [7]. Head lice infestations can be influenced by several variables, including parental education and employment status, family size, the existence of a bathroom at home, how often people bathe each week, and the use of shared personal hygiene products. Lice bites, saliva, and feces have already made the scalp itchy. The other symptoms include despair, sleeplessness, exhaustion, failure in school, mental illness, a decline in social stigma, and allergic responses. Most infestation instances go unreported because of societal shame [8].

Methodology

We searched Medline via PubMed and CENTRAL DATABASE via the Cochrane Library. The search was done using the keywords: head lice, permethrin, spinosad, pediculosis capitis, and dimethicone. Furthermore, we screened the references list of the potentially relevant studies to seek additional studies. Studies retrieved from these electronic searches and relevant references included in the bibliography of those studies were reviewed.

## Review

Diagnosis and identification

The presence of a live louse, nymph, or viable egg on the scalp or in the scalp hair is the gold standard for the diagnosis of pediculosis capitis. Using a fine-toothed lice comb with spacing between the teeth of 0.2-0.3 mm is twice as effective and four times faster than using a hand comb to find pediculosis capitis. Metal nit combs offer a higher output than plastic ones in this aspect. Additionally, moist combing has a sensitivity of 91%, making it more effective than dry combing or eye inspection for finding live lice [1]. The sensitivity of wet combing in diagnosing active infestation is better than visual inspection. Most pediculicides don't live up to their advertisements' promises of "killing lice on contact". Resistance has developed over time as a result of formulations that lack potency, slow pediculicidal action, and sublethal residue on the scalp and hair. Only malathion 0.5%, a pediculicide, continues to be effective in eliminating both lice and nits [7]. The study's geographic location, seasonal changes, population age and sex, and the male-to-female ratio of about 3:1 might all have contributed to the enormous variation in reported incidence rates [1].

Conventional treatment modalities

Permethrin Based Products

Worldwide reports of permethrin resistance have been made, and commercial pediculicides containing permethrin have also been associated with clinical failures. The prevalence of permethrin resistance in American head lice populations is high, but it is not yet consistent, and the degree of resistance is only moderately high (four to eight times) [9]. The most effective and safest drug to treat head lice infestation was permethrin 1%. Permethrin shampoo consistently performs effectively [10].

Ivermectin Shampoo

Ivermectin is frequently used as an oral therapy for infestation with louse. Systemic medicine delivery might not be necessary with topical ivermectin formulation. Ivermectin used topically prevents the negative effects associated with oral therapy. It investigated how topical ivermectin is used to treat pediculosis in individuals with various ethnic hair care practices [11]. Patients with pediculosis pubis may get oral ivermectin as a second-line therapy (with the exception of children who weigh less than 15 kg, and who shouldn't receive ivermectin). The authors also suggest oral ivermectin as a possible remedy for treating lice in the eyelashes. The use of topical ivermectin as an alternate medication for the management of pediculosis pubis is suggested in the third guideline [12].

Resistance and Treatment

One of the most often utilized methods for figuring out an insect's pyrethroid resistance is molecular analysis. Numerous molecular techniques, such as serial invasive signal amplification reaction (SISAR), quantitative multiplex sequencing melting curve analysis genotyping coupled with quantitative polymerase chain reaction (PCR), fluorescent resonance energy transfer technology (FRET), real-time PCR amplification of specific allele (rtPASA), and restriction fragment length polymorphism, have been used to identify head lice carrying resistance [13]. Due to its widespread incidence, pediculosis treatment and management are particularly crucial. Treatments often used include topical insecticides like permethrin [1%], malathion [0.5%], lindane [1%], and oral ivermectin. Initially, permethrin and lindane were both successful in curing 89.7% and 95% of head lice infections, respectively [14]. Changes in pediculicide formulations and incorrect application are two other causes of resistance. Due to essential safety modifications in formulas and unintended effects of new packaging on these goods' chemical components, the potency of pyrethrins has decreased during the past 20 years. Due to a combination of declining chemical activity and exposing head lice to diluted or insufficient dosages of pediculicides, the effectiveness of pediculicides has been significantly reduced [15]. Head lice are incredibly adaptive to their human hosts due to their architecture and physiology, yet they are also challenging to get rid of. The larvae, or nymphs, are difficult to observe due to their coloring and small size, and they are protected by several exoskeletons. A nymph can shed its exoskeleton when exposed to pediculicides, resulting in just a sublethal dosage being received. Head lice have evolved pediculicide resistance through natural selection, making infestations more difficult to eradicate [15].

Alternative Medicines

A physical treatment approach based on the idea of cold atmospheric pressure plasma (CAPP) was created to overcome the risk of resistance developing in lice. CAPPs have been created to treat a variety of ailments, from skin conditions (for example, to various cancer therapy techniques), thanks to their shown capacity to interact with biological surfaces and wounds [16]. Topical lotion containing 25% benzyl benzoate, which has to be used twice, is the gold standard treatment for head lice. The normal treatment for an infected person is to thoroughly massage 30 ml of a topical lotion containing 25% benzyl benzoate into a dry scalp until it becomes moist, leave the lotion on the affected area for 12 to 24 hours, and then wash it off with shampoo and water. The procedure is often repeated on day seven. The scalp that has been scraped or is infected may experience burning and itching as a result of this therapy [17]. One of the medicinal plants included in Iran's herbal pharmacopeia is fennel, which is widely recognized for its therapeutic benefits, including anti-nausea, digestibility, and diuretic characteristics. The fennel plant's many sections are taken into consideration when extracting the essential oil. More than 30 distinct components make up fennel essential oils, although trans-anethole, funchon, limonene, -pinene, and estragole are among the most important. The primary fennel oils have recently been demonstrated to have an insect-repellent effect in addition to having acaricidal, anti-fungal, and antibacterial effects [18].

Medications

*Lindane 1%* 

Since 1951, lindane has been accessible. It is an organochloride that is harmful to the central nervous system, and there have been several reports of severe seizures in kids who took lindane. It is offered as a 1% lindane shampoo and has to be used again every nine to 10 days, staying on the scalp for no more than four minutes. It has little ovicidal activity (between 30 and 50 percent of eggs are left unharmed), and resistance has long been noted in several regions of the world. Patients should only take it if they are unable to tolerate the first line of treatment or if their infestation has not reacted to it [3].

Permethrin 1% 

Permethrin cream rinse used for ten minutes is quite effective and well tolerated [19]. It's used on damp hair after towel-drying it after being shampooed with a non-conditioning wash. Then, it is washed off after being left on for ten minutes. Permethrin residue is left on the hair, killing any nymphs that may hatch from 20% to 30% of the eggs that weren't killed during the original treatment. The ability of permethrin to adhere to the hair shaft is hindered by conditioners and silicone-based compounds, which are included in practically all shampoos now on the market. This decreases the likelihood that permethrin will leave a residue. If live lice are found, the process must be repeated in seven to 10 days. According to contemporary recommendations, repeat therapy is most recommended on the tenth day [3].

Malathion 0.5% 

When using Malathion 0.5% for pediculosis capitis, it is essential to follow the instructions provided by the manufacturer or a healthcare professional. To use malathion 0.5% to treat head lice infestations, you should start by thoroughly combing the hair with a fine-toothed comb or lice comb to remove as many lice and nits (lice eggs) as possible. Then shake the malathion 0.5% bottle well before use and apply the malathion lotion to dry hair and scalp, ensuring complete coverage. Finally, massage the lotion into the hair and scalp and leave it on for the recommended amount of time specified on the product label or as advised by a healthcare professional. This duration can vary but is typically around eight to 12 hours. Children less than six months old and pregnant women should refrain from using malathion as mentioned in Table 1.

**Table 1:** Treatment for Pediculosis Capitis

FORMULATION	Therapeutic use	Contraindication
Permethrin based products	Residual activity and increasing drug resistance	Hypersensitivity
Ivermectin Shampoo	Neuromuscular paralysis	None
Benzyl alcohol	Non neurotoxic, non-ovicidal	Hypersensitivity
*Lindane 1%*	Ovicidal, increasing drug resistance	Premature infants, uncontrolled seizure disorders
Malathion 0.5%	Ovicidal, residual activity, increasing drug resistance	Children less than 6 months, pregnancy
Spinosad	Neuroexcitatory	None

Spinosad: an effective pediculocide

Spinosad is more practical, efficient, and doesn't need to be nit-combed. Furthermore, spinosad resistance in permethrin-resistant head lice has been observed. Spinosad crème rinse should be applied to dry hair, starting at the scalp and working your way down to the ends. After 10 minutes, the scalp and hair are washed of the product. Spinosad has no risks whether used orally, topically, intravenously, or intramuscularly. Skin absorbs it slowly and ineffectively. Contact with the eyes may irritate them, although corneal damage is uncommon. Long-term skin contact may produce a minor itch and localized redness [20]. Spinosad 0.9% primarily affects insects' nicotinic acetylcholine receptors, causing neuronal stimulation that, over prolonged durations of hyperexcitation, causes lice to become paralyzed from neuromuscular weariness. Both populations of lice that are sensitive to and resistant to permethrin are eliminated by spinosad 0.9%. It is also ovicidal, eliminating both lice and their eggs (nits) [21]. Clinical investigations have demonstrated that it is effective in preventing head lice that are permethrin-resistant. In two clinical investigations comparing the two medications, effectiveness was seen in the spinosad-treated groups at 84.6% and 86.7%, respectively, as opposed to the permethrin-treated groups (respective values of 44.9% and 42.9%; P 0.001). Clinical studies using spinosad generally showed good tolerability [22]. The pediculicidal tetracyclic macrolides spinosyn A and spinosyn D, in a 5:1 ratio, make up the natural combination known as spinosad. It is a by-product of fermentation on a nutrient-rich food source generated by bacteria of the actinomycetes genus Saccharopolyspora spinosa. It has been used as a pesticide for a very long time and is considered a reduced-risk pesticide product by the US Environmental Protection Agency [20].

Both parents and childcare workers have access to information on pediculosis capitis, although uptake is unclear. The majority of parents also choose to conceal the infestation in their kids out of embarrassment or to avoid social stigma, failing to alert kindergarten instructors and health coordinators to the issue. For the most vulnerable populations, most notably kindergarten students, our findings have implications for future practice and the deployment of specific public health initiatives [23]. The most frequent form of therapy is pediculicide topical application. Although frequently employed, substances having a neurotoxic mechanism of action are losing their efficacy due to resistant parasite populations. Aside from that, safety issues limit their use. Dimeticones, silicone oils that tend to properly coat surfaces and have a low surface tension, only have a physical mechanism of action. There is little chance that head lice will develop resistance to this class of chemicals because they are harmless and extremely efficient. Active contact tracking of contacts and coordinated pediculicide treatment are necessary for epidemic control [24]. Natural oils are increasingly being used for both prevention and therapy. More encouragement is needed to use pediculicides or non-pharmacological treatments for pediculosis capitis infestation in a responsible manner [25]. Along with clear treatment strategies, proper parental education is a crucial part of treating head lice. Currently, oral medication, comb usage, and topical pediculicides are the three alternatives mentioned for treating head lice. Permethrin and other pyrethroids, synergized pyrethrins, and malathion are the most popular topical pediculicides among those made available in Italy [26].

## Conclusions

A number of topical pediculicides can be used to treat pediculosis capitis. For safety concerns, some of these pediculicides should not be used, especially on children under the age of two. The choice of a pediculosis medication should take into account cost, local patterns of resistance, effectiveness, and safety. Resistance to certain of these pediculicides, especially those having a neurotoxic mode of action, is another concern that could limit their usage. It is important to assess the effectiveness and safety of new goods. Wet combing takes a lot of time, so it shouldn't be the only intervention utilized by the general populace. In the event that treatment is unsuccessful, it is important to take into account potential misdiagnosis (no active infestation or misidentification), lack of adherence (patient unable or unwilling to adhere to treatment protocol), insufficient treatment (using insufficient product to saturate hair), reinfestation (lice acquired after treatment), lack of ovicidal or residual killing properties of the pediculicide (eggs not killed can hatch and cause self-reinfestation), and/or lice resistance to treatment. 
